# Precise Regulation of Interlayer Stacking Modes in Trinuclear Copper Organic Frameworks for Efficient Photocatalytic Reduction of Uranium(VI)

**DOI:** 10.1002/advs.202406530

**Published:** 2024-09-27

**Authors:** Zhi Gao, Sijia Lv, Yue Wang, Zhenzhen Xu, Yingtong Zong, Yuan Tao, Yingji Zhao, Xingyu Liu, Shuhui Yu, Mingbiao Luo, Nithima Khaorapapong, Ruikang Zhang, Yusuke Yamauchi

**Affiliations:** ^1^ Jiangxi Province Key Laboratory of Functional Organic Polymers East China University of Technology Nanchang Jiangxi 330013 China; ^2^ College of Chemistry and Chemical Engineering Gannan Normal University Ganzhou Jiangxi 341000 China; ^3^ MOE Key Laboratory of Bioinorganic and Synthetic Chemistry/KLGHEI of Environment and Energy Chemistry School of Chemistry Sun Yat‐Sen University Guangzhou Guangdong 510275 China; ^4^ Department of Materials Process Engineering Graduate School of Engineering Nagoya University Nagoya 464‐8603 Japan; ^5^ Materials Chemistry Research Center Department of Chemistry and Center of Excellence for Innovation in Chemistry Faculty of Science Khon Kaen University Khon Kaen 40002 Thailand; ^6^ College of Chemistry and Materials Science Hebei Normal University Shijiazhuang Hebei 050024 China; ^7^ Department of Chemical and Biomolecular Engineering Yonsei University 50 Yonsei‐ro Seodaemun‐gu Seoul 03722 South Korea; ^8^ Australian Institute for Bioengineering and Nanotechnology (AIBN) The University of Queensland Brisbane QLD 4072 Australia

**Keywords:** copper organic frameworks, electron–hole separation efficiency, interlayer stacking, photocatalytic U(VI) reduction, radioactive wastewater

## Abstract

The interlayer stacking modes of 2D covalent‐organic frameworks (COFs) directly influence their structural features, ultimately determining their functional output. However, controllably modulating the interlayer stacking structure in traditional 2D metal‐free COFs, based on the same building blocks, remains challenging. Here, two trinuclear copper organic frameworks are synthesized successfully with different interlayer stacking structures: eclipsed AA stacking in Cu_3_‐PA‐COF‐AA and staggered ABC stacking in Cu_3_‐PA‐COF‐ABC, using the same monomers. Remarkably, various functionalities, including porosity and electronic and optical properties, can be effectively regulated by interlayer stacking. As a result, Cu_3_‐PA‐COF‐AA and Cu_3_‐PA‐COF‐ABC exhibit significantly different activities toward the photoreduction of U(VI), presenting a promising strategy for removing radioactive uranium pollution. Due to its broader visible‐light absorption range and superior photogenerated carrier migration and separation efficiency, Cu_3_‐PA‐COF‐AA achieves a U(VI) removal ratio of 93.6% without additional sacrificial agents in an air atmosphere—≈2.2 times higher than that of Cu_3_‐PA‐COF‐ABC (42.0%). To the best of the knowledge, this is the first study to elucidate the effect of interlayer stacking in COFs on the photocatalytic activity of U(VI) reduction. This finding may inspire further exploration of the structure‐function relationship in COFs as photocatalysts and their potential for photoinduced removal of radionuclides.

## Graphical abstract

Without altering building blocks, two trinuclear copper organic frameworks with different interlayer stacking structures (eclipsed AA stacking and staggered ABC stacking) are successfully synthesized. As a result, the structural and photoelectric properties are significantly regulated by the interlayer stacking. The photocatalytic activity of Cu_3_‐PA‐COF‐AA toward U(VI) reduction is much superior to that of Cu_3_‐PA‐COF‐ABC.

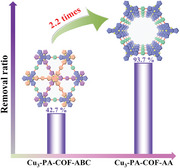

## 1 Introduction

The rapid development of nuclear energy has led to an increasing demand for uranium, which serves as the principal ingredient in most nuclear power plants.^[^ ^1^ ^]^ However, uranium mining inevitably generates large quantities of radioactive, uranium‐containing wastewater.^[^ ^2^ ^]^ The high chemical toxicity and radioactivity of highly soluble U(VI) in wastewater pose a serious threat to human health,^[^ ^3^ ^]^ thereby drawing widespread attention to the need for reasonable and safe disposal of nuclear waste. Up to now, several technologies have been proposed to effectively remove U(VI) such as solvent extraction,^[^ ^4^ ^]^ membrane separation,^[^ ^5^ ^]^ adsorption,^[^ ^6^ ^]^ photocatalytic reduction,^[^ ^7^ ^]^ and so on. Among these, photocatalytic reduction technology is widely regarded as one of the most promising pathways to remove U(VI) pollution, due to its remarkable advantages of high efficiency, ease of operation, and low energy consumption.^[^ ^8^ ^]^ This technology leverages the solubility difference between soluble U(VI) and insoluble U(IV).^[^ ^9^ ^]^

Covalent‐organic frameworks (COFs), as emerging porous crystalline materials, have garnered significant attention in the field of photocatalysis due to their high stability, rich porosity, and easily tunable periodic skeletons.^[^ ^10^ ^]^ Currently, several COF‐based photocatalysts have been designed for the photocatalytic reduction of U(VI) to U(IV).^[^ ^11^ ^]^ The incorporation of binding sites, such as amidoxime groups, into the COF framework to selectively bind U(VI) and subsequently drive U(VI) photoreduction is widely considered an effective strategy to eliminate soluble U(VI).^[^ ^12^ ^]^ In addition, linkage engineering has also been proposed to enhance the U(VI) photoreduction efficiency of COFs.^[^ ^13^ ^]^ For example, Qiu et al. designed a highly stable C═C linkage instead of a dynamic C═N bond in the COF skeleton, which endows the COFs with a fully extended π‐conjugated structure, thereby improving photocatalytic activity. Furthermore, by taking advantage of the abundant variability of building blocks, the electronic and optical properties of COFs, such as bandgap, charge transport, and separation efficiency, can also be regulated to achieve high‐efficiency U(VI) photoreduction.^[^ ^14^ ^]^ Although the aforementioned strategies have achieved promising results, the effect of intrinsic structural properties of COFs, such as interlayer stacking mode, on U(VI) photoreduction activity has not been explored. This may provide an alternative and inspiring approach to enhance photocatalytic activity.

Similar to 2D materials such as graphene^[^ ^15^ ^]^ and transition metal dichalcogenides,^[^ ^16^ ^]^ the interlayer stacking structures of 2D COFs not only undoubtedly affect structural features such as crystallinity and porosity^[^ ^17^ ^]^ but also determine their electronic and optical properties.^[^ ^18^ ^]^ These properties, in turn, play a prominent role in U(VI) photoreduction performance. However, the controllable synthesis of traditional pure organic COFs with variable interlayer stacking using the same building units is very difficult to achieve. Recently, a copper organic framework synthesized using Cu^I^ cyclic trinuclear unit (Cu_3_) as a secondary building block has received considerable attention.^[^ ^19^ ^]^ The trinuclear Cu_3_ clusters can provide additional metallophilic interactions compared to pure organic building blocks,^[^ ^20^ ^]^ which are beneficial for regulating the interlayer stacking mode. This unique property of copper organic frameworks may provide a promising platform to explore how interlayer stacking modes in 2D COFs impact U(VI) photoreduction activity.

In this work, two copper organic frameworks with different interlayer stacking modes (eclipsed AA stacking in Cu_3_‐PA‐COF‐AA and staggered ABC stacking in Cu_3_‐PA‐COF‐ABC) are successfully synthesized using the identical building blocks of *p*‐phenylenediamine (PA) and Cu_3_(PyCA)_3_·H_2_O (Cu_3_, 1H‐PyCA = 1H‐pyrazole‐4‐carbaldehyde). As expected, the structural and photoelectric properties are significantly influenced by the interlayer stacking modes. The Cu_3_‐PA‐COF‐AA exhibits stronger light absorption capacity, more efficient photogenerated charge separation efficiency, and better photoreduction U(VI) activity compared to Cu_3_‐PA‐COF‐ABC. Under visible light irradiation, Cu_3_‐PA‐COF‐AA achieves a U(VI) removal efficiency of 93.6% with superior reusability without the usage of sacrificial agents. This work provides a new strategy to enhance the U(VI) photoreduction activity of COFs and presents a promising solution for addressing U(VI) pollution in actual radioactive wastewater.

## 2 Results and Discussion

### 2.1 Design, Synthesis, and Characterization of COFs

**Scheme 1:**
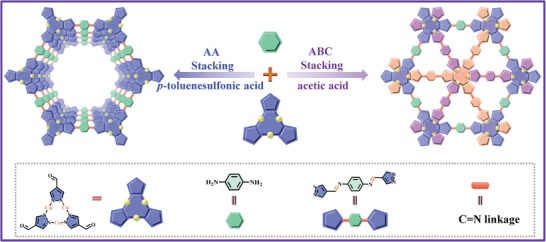
Schematic representation for controllable synthesis of trinuclear copper organic framework with different interlayer stacking structures.

The Cu_3_ monomer was prepared through a solvothermal reaction of Cu(NO_3_)_2_·3H_2_O with 1H‐PyCA at 100 °C for 24 h according to the previously reported method.^[^ ^21^ ^]^ After the reaction, the light yellow crystals of Cu_3_ were observed under an optical microscope (Figure S1, Supporting Information). The powder X‐ray diffraction (PXRD) pattern of the as‐prepared Cu_3_ cluster is in good agreement with the reported pattern (Figure S2, Supporting Information),^[^ ^22^ ^]^ indicating successful synthesis. Fourier transform infrared (FT‐IR) spectra of 1H‐PyCA and Cu_3_ were compared. The C═O stretching vibration at 1686 cm^−1^ in 1H‐PyCA^[^ ^21^ ^]^ shifts to 1652 cm^−1^ in the Cu_3_ building block (Figure S3, Supporting Information), indicating the successful coordination of Cu to N sites. Then, the Cu_3_‐PA‐COF‐AA and Cu_3_‐PA‐COF‐ABC with different interlayer stacking modes were prepared using the identical building units of PA and Cu_3_ (**Scheme** **1**). The solvothermal method using acetic acid as a catalyst was utilized to obtain Cu_3_‐PA‐COF‐ABC. To transform the stacking mode from ABC to AA, the mechanochemical synthesis and *p*‐toluenesulfonic acid instead of acetic acid as catalyst was carefully optimized to fabricate Cu_3_‐PA‐COF‐AA.^[^ ^23^ ^]^

**Figure 1:**
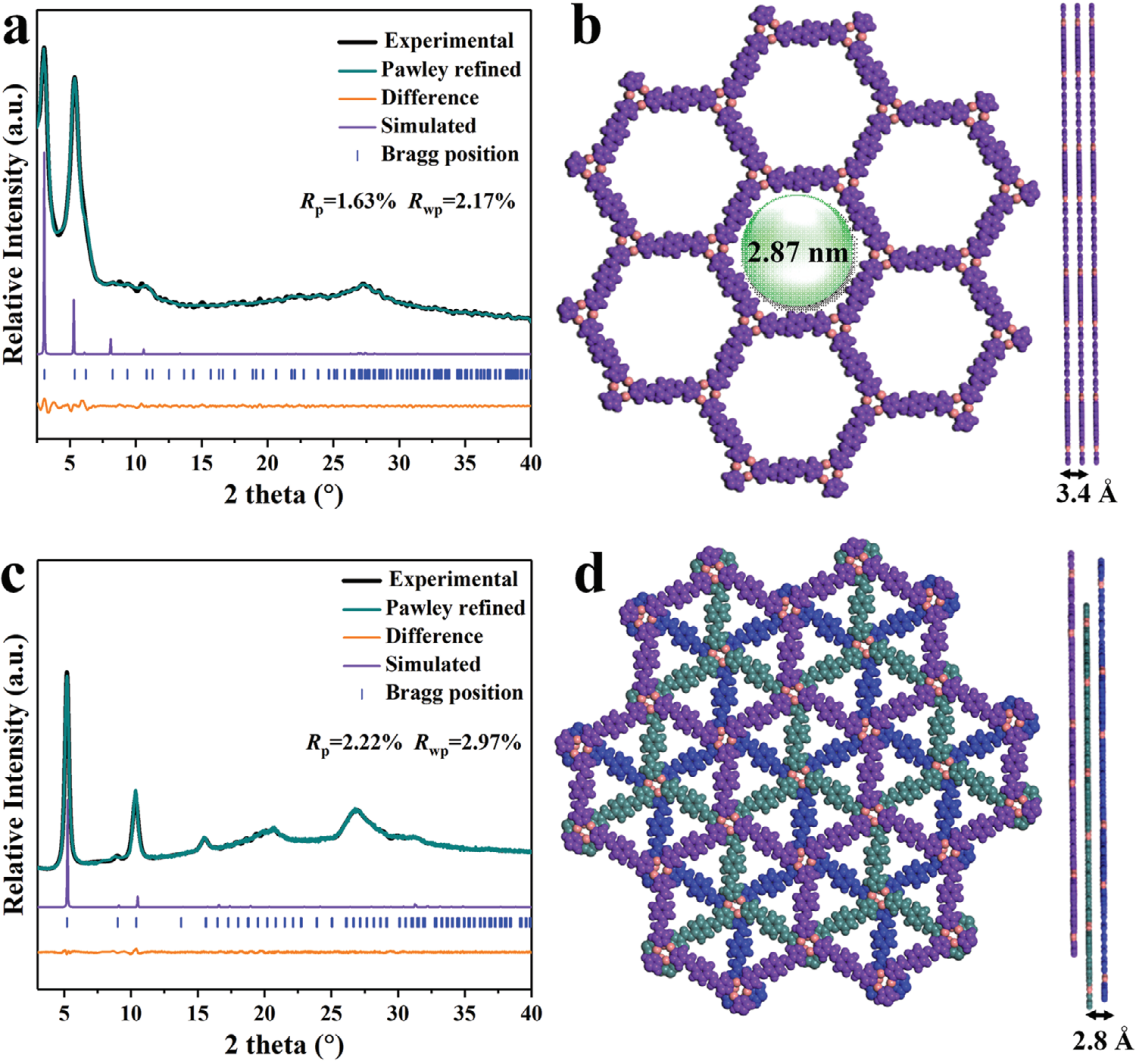
PXRD structural analysis of Cu_3_‐PA‐COF‐AA (a) and Cu_3_‐PA‐COF‐ABC (c); Refined crystal structure of Cu_3_‐PA‐COF‐AA (b) and Cu_3_‐PA‐COF‐ABC (d).

The PXRD measurements in combination with the theoretical structural simulations were carried out to assess the crystallinity of Cu_3_‐PA‐COF‐AA and Cu_3_‐PA‐COF‐ABC. The experimental PXRD patterns confirm that both are highly crystalline porous polymers (**Figure** **1**a,c). The *P*6 and $R\bar{3}$ space groups were constructed for Cu_3_‐PA‐COF‐AA and Cu_3_‐PA‐COF‐ABC, respectively. Pawley refinement of the PXRD patterns was performed for full‐profile fitting based on the proposed models using Materials Studio Software, yielding the lattice parameters of *a* = *b* = 32.811 Å, *c* = 3.405 Å, and *α* = *β* = 90°, *γ* = 120° in Cu_3_‐PA‐COF‐AA, as well as *a* = *b* = 34.072 Å, c = 5.771 Å, and *α* = *β* = 90°, *γ* = 120° in Cu_3_‐PA‐COF‐ABC. The simulated PXRD patterns based on the eclipsed stacking model (AA stacking, Figure 1b) and staggered stacking model (ABC stacking, Figure 1d) closely match the experimentally measured patterns of Cu_3_‐PA‐COF‐AA (Figure 1a) and Cu_3_‐PA‐COF‐ABC (Figure 1c), respectively. This is supported by the negligible difference curves with low unweighted‐profile *R* factor (*R*_p_) of 1.63% in Cu_3_‐PA‐COF‐AA and 2.22% in Cu_3_‐PA‐COF‐ABC, as well as low weighted profile *R* factor (*R*_wp_) of 2.17% for Cu_3_‐PA‐COF‐AA and 2.97% for Cu_3_‐PA‐COF‐ABC. The typical diffraction peaks at 3.06°, 5.37°, 6.21°, 8.24°, 9.35° and 10.8° assigned to the (100), (110), (200), (210), (300), and (220) facets were detected in Cu_3_‐PA‐COF‐AA, respectively. The strong peak at 3.06° associated with the (100) plane indicates the regularly ordered porosity of Cu_3_‐PA‐COF‐AA. The broad peak at 27.3° is attributed to the diffraction peak of the (001) plane, associated with π‐stacking. In the case of Cu_3_‐PA‐COF‐ABC, the characteristic peaks of the (110), (220), (330), (321), (440), and (621) planes were observed at 5.20°, 10.38°, 15.58°, 20.17°, 20.81° and 26.63°, respectively.

**Figure 2:**
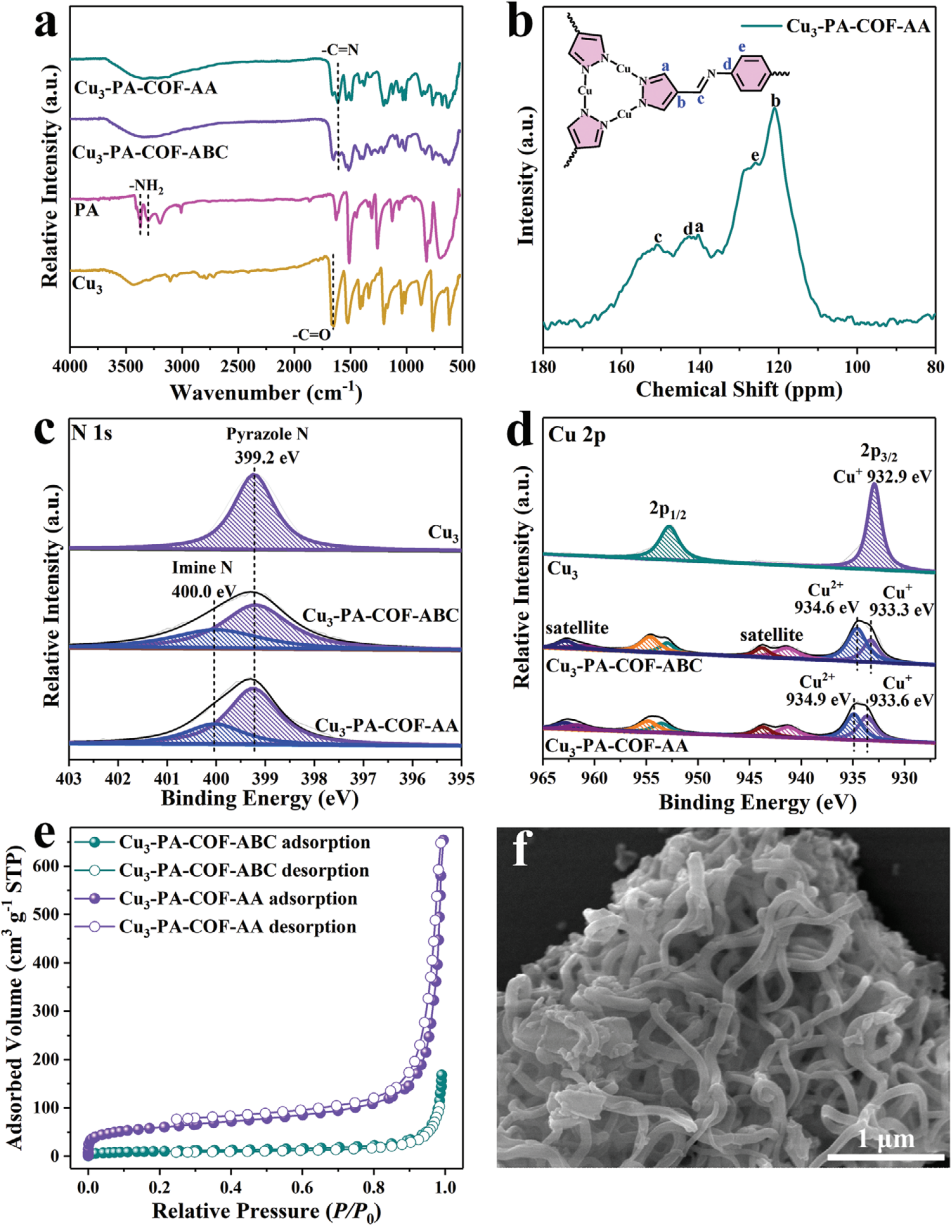
FT‐IR spectra (a); Solid‐state NMR spectrum (b); XPS spectra of N 1s (c) and Cu 2p (d); N_2_ sorption isotherms (e); SEM image of Cu_3_‐PA‐COF‐AA (f).

The chemical structures of the two COFs were confirmed by FT‐IR and solid‐state nuclear magnetic resonance (NMR) analysis. First, the FT‐IR spectra of two COFs were compared with those of the Cu_3_ and PA building units (**Figure** **2**a). PA exhibits an N─H stretching vibration at 3400‐3300 cm^−1^,^[^ ^19a^ ^]^ which disappears in Cu_3_‐PA‐COF‐AA and Cu_3_‐PA‐COF‐ABC. The intensity of the C═O stretching vibration in two COFs is much weaker than that in the Cu_3_ cluster. Notably, a new peak at ≈1610 cm^−1^ assigned to the C═N stretching band was detected in two COFs.^[^ ^24^ ^]^ The FT‐IR results demonstrate the formation of imine linkages in Cu_3_‐PA‐COF‐AA and Cu_3_‐PA‐COF‐ABC by the Schiff‐base condensation reaction between Cu_3_ and PA monomers. As verified by the ^13^C CP/MAS NMR spectra (Figures 2b and S4, Supporting Information), both Cu_3_‐PA‐COF‐AA and Cu_3_‐PA‐COF‐ABC exhibit a characteristic resonance peak of imine carbons at ≈150 ppm,^[^ ^25^ ^]^ further confirming the formation of imine linkage in two COFs. The X‐ray photoelectron spectroscopy (XPS) measurements of Cu_3_, Cu_3_‐PA‐COF‐AA, and Cu_3_‐PA‐COF‐ABC were carried out. The XPS survey spectra indicate the presence of C, N, O, and Cu elements in these materials (Figure S5, Supporting Information). The high‐resolution XPS spectra of N 1s were analyzed. Cu_3_ building block exhibits a single peak centered at 399.2 eV assigned to pyrazole N (Figure 2c). In contrast, a new signal related to imine N at the binding energy of ≈400.0 eV was detected in both COFs. In the Cu 2p spectra (Figure 2d), sharp and symmetrical peaks of Cu 2p_1/2_ and 2p_3/2_ at 952.8 and 932.9 eV were observed without satellite peaks, revealing the presence of only Cu^+^ species in Cu_3_ cluster.^[^ ^19d^ ^]^ In contrast, Cu_3_‐PA‐COF‐AA and Cu_3_‐PA‐COF‐ABC exhibit asymmetrical Cu 2p signals, which were deconvoluted into two components of Cu^+^ and Cu^2+^ species. The appearance of Cu^2+^ species is attributed to the partial oxidation of Cu^+^, which does not destroy the cyclic triangular skeleton of the Cu_3_ building block.^[^ ^19b^ ^]^ Notably, the binding energy of Cu^+^ species in Cu_3_‐PA‐COF‐AA (933.6 eV) and Cu_3_‐PA‐COF‐ABC (933.3 eV) is higher than in the Cu_3_ monomer (932.9 eV), indicating electron transfer from Cu_3_ to PA, which is attributed to the formation of an internal electric field within the COF framework.^[^ ^26^ ^]^ The highest Cu^+^ binding energy in Cu_3_‐PA‐COF‐AA indicates the strongest internal electric field within the skeleton, confirming the significant potential of Cu_3_‐PA‐COF‐AA as a photocatalyst for driving photocatalytic reactions. Additionally, the XPS spectra of C and O elements are presented in Figures S6 and S7 (Supporting Information).

N_2_ sorption isotherms at 77 K were recorded to examine the surface area and porous structure (Figure 2e). The Brunauer–Emmett–Teller (BET) surface area estimated in Cu_3_‐PA‐COF‐AA (496.6 m^2^ g^−1^) is much higher than that in Cu_3_‐PA‐COF‐ABC (24.56 m^2^ g^−1^). The Cu_3_‐PA‐COF‐AA shows a narrow pore size distribution with an average pore width of 2.82 nm (Figure S8, Supporting Information), agreeing well with the theoretical values obtained from the AA eclipse stacking (2.87 nm). These results suggest that AA eclipse stacking leads to a larger surface area and pore size, which facilitates the transport and adsorption of reactants toward active sites in Cu_3_‐PA‐COF‐AA during photocatalytic reactions. As revealed by the thermogravimetric analysis in Figure S9 (Supporting Information), two COFs can keep high stability up to 280 °C in air atmosphere. Moreover, the Cu_3_‐PA‐COF‐AA and Cu_3_‐PA‐COF‐ABC also exhibit good chemical stability in aqueous acid (pH 2.0) and alkaline (pH 12.0) solutions for 24 h, as well as excellent radiation resistance ability exposed in 600 kGy *β*‐ray (Figure S10, Supporting Information). From scanning electron microscopy (SEM) images, Cu_3_‐PA‐COF‐AA and Cu_3_‐PA‐COF‐ABC show nanofiber morphology (Figures 2f and S11, Supporting Information), which is different from the needle‐shaped structure of the Cu_3_ cluster (Figure S1, Supporting Information). The high‐resolution transmission electron microscopy (HRTEM) image of Cu_3_‐PA‐COF‐AA demonstrates the lattice fringes with spacing ≈0.35 nm (Figure S12, Supporting Information), which is close to the calculated interlayer distance in Figure 1b, verifying the π‐π stacking.

### 2.2 Optoelectronic Properties

**Figure 3:**
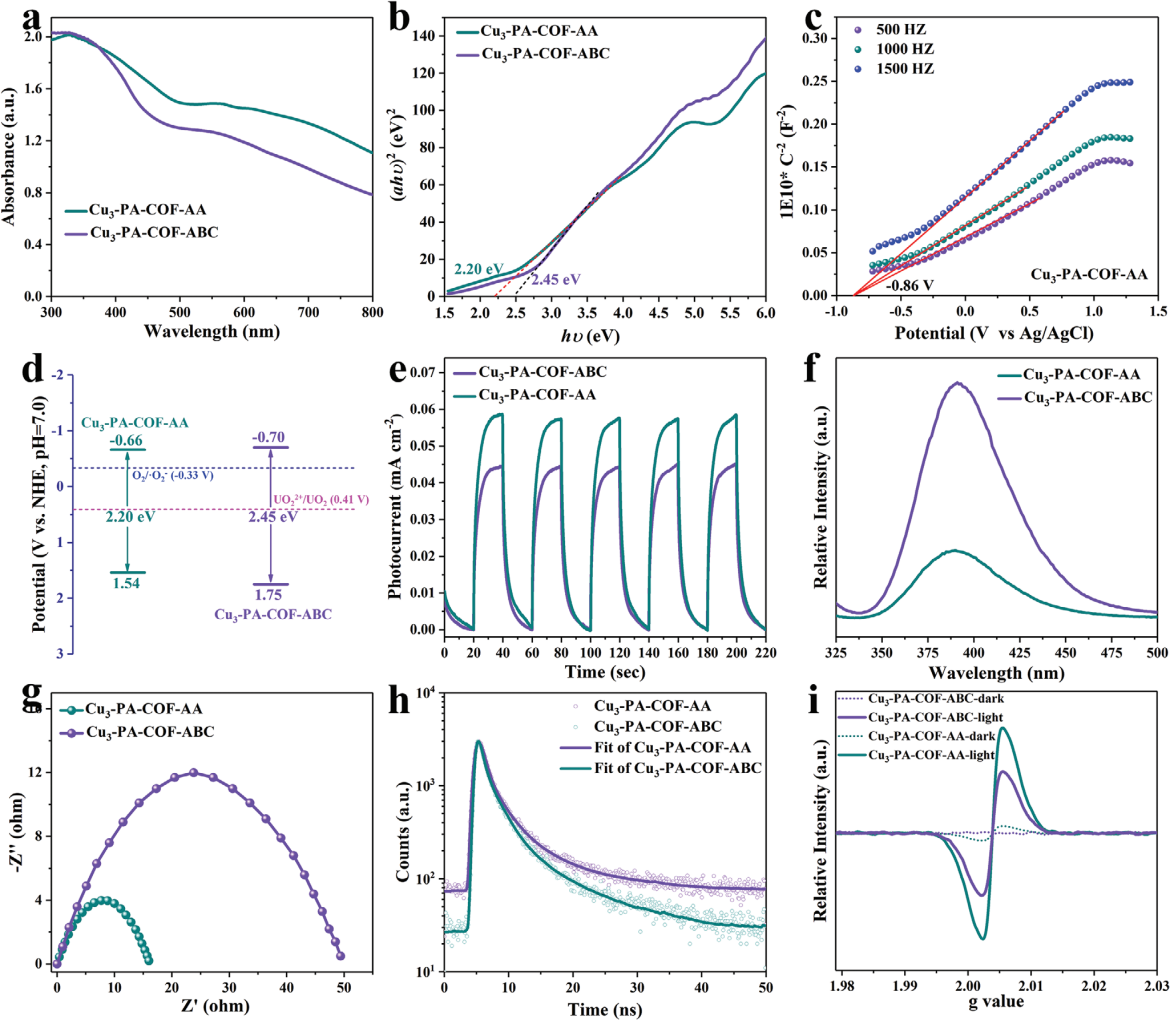
UV–vis DRS spectra (a); Tauc plot for bandgap calculation (b); Mott‐Schottky plots (c) Band structure alignments (d); Transient current density (e); Steady‐state PL spectra (f); Nyquist plots (g); Time‐resolved PL decay spectra (h); EPR conduction band electron spectra in the dark and upon visible light irradiation (i).

UV–vis diffuse reflectance spectroscopy (UV–vis DRS) shows that Cu_3_‐PA‐COF‐AA and Cu_3_‐PA‐COF‐ABC exhibit significant visible‐light absorption extending to 800 nm (**Figure** **3**a). Notably, Cu_3_‐PA‐COF‐AA exhibits greater visible‐light absorption than Cu_3_‐PA‐COF‐ABC, which can be attributed to more effective π‐electron conduction in the eclipsed AA stacking compared to the staggered ABC stacking.^[^ ^27^ ^]^ Furthermore, the optimal bandgap (*E*_g_) was calculated using the Tauc plot method. As shown in Figure 3b, the *E*_g_ of Cu_3_‐PA‐COF‐AA (2.20 eV) is narrower than that of Cu_3_‐PA‐COF‐ABC (2.45 eV), which can be ascribed to the stronger π–π and Cu–Cu interactions between layers in Cu_3_‐PA‐COF‐AA.^[^ ^14a^ ^]^ The narrower *E*_g_ in Cu_3_‐PA‐COF‐AA indicates that the least energy is required to drive the photocatalytic reaction. Electrochemical Mott‐Schottky measurements were carried out to determine the flat band potential and then further reveal the energy band structure. Positive slopes were obtained for Cu_3_‐PA‐COF‐AA and Cu_3_‐PA‐COF‐ABC (Figure 3c and S13, Supporting Information), characteristic of typical *n*‐type semiconductors. Since the flat band potential is close to the conduction band (CB) position in *n*‐type semiconductors,^[^ ^28^ ^]^ the *E*_CB_ of Cu_3_‐PA‐COF‐AA and Cu_3_‐PA‐COF‐ABC were determined to be −0.86 and −0.90 V versus Ag/AgCl (corresponding to −0.66 and −0.70 V vs NHE). According to the equation of *E*_g_ = *E*_VB_ – *E*_CB_, the *E*_VB_ was calculated to be 1.54 and 1.75 V versus NHE for Cu_3_‐PA‐COF‐AA and Cu_3_‐PA‐COF‐ABC, respectively. Based on these results, the corresponding band structure alignments were plotted in Figure 3d.^[^ ^29^ ^]^ It is worth noting that the *E*_CB_ values in both samples are more negative than the U(VI)/U(IV) redox potential (0.41 V vs NHE), demonstrating the thermodynamic feasibility of the photocatalytic reduction of U(VI) to U(IV).^[^ ^30^ ^]^ In addition, the cyclic voltammetry (CV) measurements were carried out. As shown in Figure S14 (Supporting Information), Cu_3_‐PA‐COF‐AA exhibits the onset oxidation potential at ≈−0.61 V versus Ag/AgCl, which is much lower than the reduction potential of U(VI)/U(IV) (0.41 V vs NHE), further demonstrating that the reduction of U(VI) by Cu_3_‐PA‐COF‐AA is thermodynamically feasible. The above results also indicate that the structural properties of Cu_3_‐PA‐COF‐AA and Cu_3_‐PA‐COF‐ABC are similar to those reported (Table S1, Supporting Information).^[^ ^21^, ^22^ ^]^

Transient photocurrent response and photoluminescence (PL) measurements were conducted to assess charge‐transfer behaviors.^[^ ^31^ ^]^ As shown in Figure 3e, the transient current density of Cu_3_‐PA‐COF‐AA is ≈1.3 times higher than that of Cu_3_‐PA‐COF‐ABC. The PL intensity of Cu_3_‐PA‐COF‐AA is significantly lower than that of Cu_3_‐PA‐COF‐ABC (Figure 3f). These results indicate that Cu_3_‐PA‐COF‐AA with eclipsed AA stacking more effectively enhances the spatial separation of photogenerated charge carriers and prevents electron–hole recombination compared to Cu_3_‐PA‐COF‐ABC with staggered ABC stacking. Moreover, the Nyquist curves obtained from electrochemical impedance spectroscopy (EIS) measurements reveal that the semicircle diameter of Cu_3_‐PA‐COF‐AA is much smaller than that of Cu_3_‐PA‐COF‐ABC (Figure 3g), implying the lower interfacial charge transport resistance in Cu_3_‐PA‐COF‐AA,^[^ ^32^ ^]^ which will, in turn, promote the charge transfer and thus improve photocatalytic activity. In addition, the specific charge carrier dynamics were explored by the time‐resolved PL decay spectroscopy. The average PL lifetime of Cu_3_‐PA‐COF‐AA (3.96 ns) is longer than that of Cu_3_‐PA‐COF‐ABC (3.74 ns). This is directly evidenced by the PL decay curves in Figure 3h, which reflect a lower PL decay rate in Cu_3_‐PA‐COF‐AA, indicating a longer lifetime of photogenerated charges on the surface of Cu_3_‐PA‐COF‐AA for photocatalytic reactions.^[^ ^33^ ^]^ Electron paramagnetic resonance (EPR) measurements were conducted to assess the formation of visible‐light‐induced charge. As depicted in Figure 3i, under visible light irradiation, the more obvious signals at about g = 2.004 were found in both Cu_3_‐PA‐COF‐AA and Cu_3_‐PA‐COF‐ABC compared to those in the dark, which is assigned to the formation of light‐induced conduction band electrons.^[^ ^30^ ^]^ Notably, the signal intensity of Cu_3_‐PA‐COF‐AA is significantly stronger than that of Cu_3_‐PA‐COF‐ABC, indicating a greater ability of Cu_3_‐PA‐COF‐AA to generate charge carriers. Taken together, these results demonstrate the significant potential of Cu_3_‐PA‐COF‐AA as a novel photocatalyst platform for the photocatalytic reduction of U(VI)‐containing wastewater to U(IV) precipitate due to its intriguing structural features (well‐defined framework, high stability, and rich porosity) and excellent photoelectric properties (effective visible light‐harvesting ability, suitable bandgap, and outstanding charge carrier separation and transport efficiency). As a proof‐of‐concept, a series of photocatalytic experiments were subsequently performed to evaluate the feasibility of Cu_3_‐PA‐COF‐AA for the photoreduction of U(VI) to U(IV).

### 2.3 Photocatalytic Reduction of U(VI)

**Figure 4:**
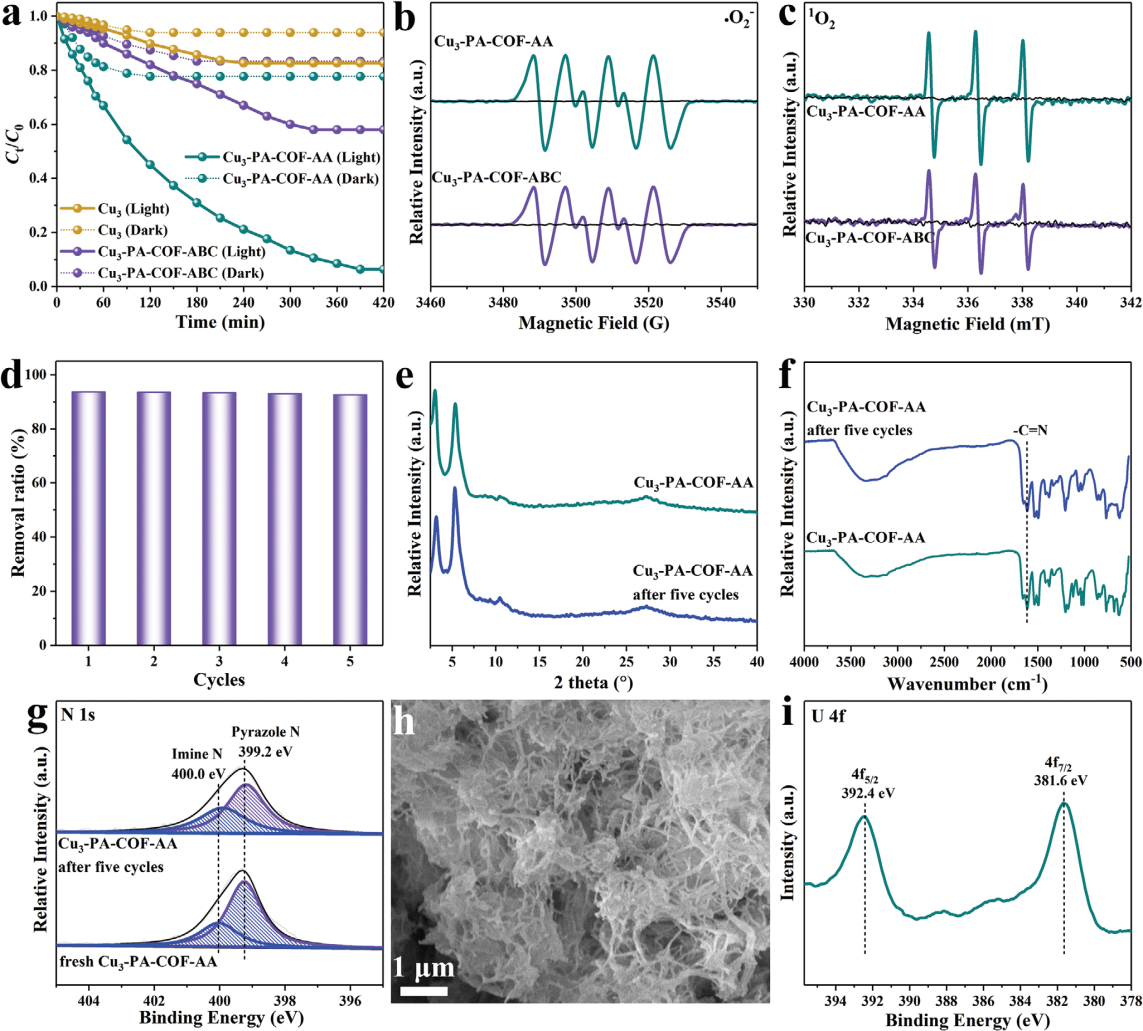
U(VI) removal ratio in the dark and upon visible‐light irradiation (a); EPR spectra of •O_2_^−^ (b) and ^1^O_2_ radicals (c); U(VI) removal ratio of Cu_3_‐PA‐COF‐AA over five successive cycles (d); XRD patterns (e); FT‐IR spectra (f); XPS of N 1s spectra (g); SEM image of Cu_3_‐PA‐COF‐AA after five cycles (h); XPS of U 4f spectrum for Cu_3_‐PA‐COF‐AA after reaction (i).

The photocatalytic activity was initially explored in the U(VI)‐spiked groundwater (20 ppm) without any sacrificial reagents in the air atmosphere with a solid‐to‐liquid ratio of 0.05 g L^−1^. As shown in **Figure** **4**a, in the dark, Cu_3_‐PA‐COF‐AA exhibits stronger adsorption ability than Cu_3_‐PA‐COF‐ABC, though both demonstrate a low U(VI) removal ratio. Upon visible‐light irradiation, the U(VI) removal ratio in Cu_3_‐PA‐COF‐AA sharply increases from 22.3% to 93.6%, which is ≈2.2 times higher than that of Cu_3_‐PA‐COF‐ABC (42.0%), demonstrating outstanding photoinduced U(VI) removal ability. The U(VI) removal capacity in Cu_3_‐PA‐COF‐AA reaches 375 mg g^−1^ after 390 min of irradiation (Figure S15, Supporting Information). Furthermore, the photoreaction rate constant (*k*) at 25 °C was calculated to determine the reaction kinetics,^[^ ^34^ ^]^ which follows a pseudo‐first‐order kinetics model (Figure S16, Supporting Information). The *k* calculated in Cu_3_‐PA‐COF‐AA (6.80 × 10^−3^ min^−1^) is ≈4.1 times higher than that in Cu_3_‐PA‐COF‐ABC (1.67 × 10^−3^ min^−1^). This high U(VI) removal ability, achieved under a low solid‐to‐liquid ratio, enables Cu_3_‐PA‐COF‐AA to outperform the most recently reported photocatalysts (Table S2, Supporting Information). Additionally, the photocatalytic reduction activity of Cu_3_‐PA‐COF‐AA was explored under the N_2_ atmosphere (Figure S17, Supporting Information), and the U(VI) removal ratio was further improved to 98.7%.

EPR experiments were performed to further investigate the radicals produced by Cu_3_‐PA‐COF‐AA and Cu_3_‐PA‐COF‐ABC during the photocatalytic process. As shown in Figure 4b, no EPR signals were observed in the dark. However, under visible light irradiation, characteristic signals corresponding to superoxide radicals (•O_2_^−^) were detected in both COFs, as the *E*_CB_ in them is more negative than the O_2_/•O_2_^−^ potential (−0.33 V vs NHE) (Figure 3d). However, signals corresponding to hydroxyl radicals (•OH) were not observed under visible light irradiation, due to the lower *E*_VB_ value compared to the •OH/H₂O potential (2.68 V vs NHE). Therefore, Cu_3_‐PA‐COF‐AA and Cu_3_‐PA‐COF‐ABC can effectively reduce dissolved O_2_ to generate •O_2_^−^, while the oxidation of H_2_O to •OH is difficult to achieve. Notably, the •O_2_^−^ signal in Cu_3_‐PA‐COF‐AA is stronger than in Cu_3_‐PA‐COF‐ABC, indicating the superior stability of Cu_3_‐PA‐COF‐AA in generating •O_2_^−^ for U(VI) photoreduction. Moreover, the generation of singlet oxygen (¹O_2_) in Cu_3_‐PA‐COF‐AA and Cu_3_‐PA‐COF‐ABC under visible light irradiation indicates that both are highly electron‐enriched (Figure 4c).^[^ ^35^ ^]^ The stronger signal in Cu_3_‐PA‐COF‐AA reflects its superior electron‐enriching ability,^[^ ^36^ ^]^ which is beneficial for the photocatalytic reduction of U(VI) on the catalyst surface. Scavenger measurements were conducted to further confirm the active species in Cu_3_‐PA‐COF‐AA during the photocatalytic reduction of U(VI). Silver nitrate and *p*­benzoquinone were used to selectively eliminate electron and •O_2_^−^, respectively. As shown in Figure S18 (Supporting Information), the U(VI) removal ratio of Cu_3_‐PA‐COF‐AA significantly decreases from 93.6% to 50.2% and 63.1% after the addition of silver nitrate and *p*­benzoquinone, respectively. This conclusively demonstrates that the electron and •O_2_^−^ are crucial active species during photoreduction U(VI) in Cu_3_‐PA‐COF‐AA.

The stability and reusability, crucial properties of photocatalysts, were evaluated over five successive reaction rounds. The U(VI) removal ratio in Cu_3_‐PA‐COF‐AA after five catalytic cycles remains nearly identical to the initial value, demonstrating superior reusability (Figure 4d). The diffraction peaks and intensities of the XRD pattern in Cu_3_‐PA‐COF‐AA after five cycles are nearly the same as the initial pattern, with no additional characteristic peaks (Figure 4e), confirming the preservation of the initial framework structure. Additionally, the stretching band of the imine linkage is also well maintained as demonstrated by the FT‐IR spectra in Figure 4f. The XPS spectra of N 1s reveal that the typical imine N peak in Cu_3_‐PA‐COF‐AA is still detectable after five cycles (Figure 4g). The unchanged XPS spectra of O 2p and C 1s are presented in Figures S19 and S20 (Supporting Information). Furthermore, the SEM image reveals a negligible change in nanofiber morphology (Figure 4h). These characterization results demonstrate the strong photochemical stability of Cu_3_‐PA‐COF‐AA. Additionally, as demonstrated by the XPS survey spectra (Figure S21, Supporting Information), the U element was found in Cu_3_‐PA‐COF‐AA after five cycles, which was not detected in the initial Cu_3_‐PA‐COF‐AA. Furthermore, the high‐resolution U 4f spectrum was analyzed, showing binding energies of U 4f_7/2_ and U 4f_5/2_ signals at ≈381.6 and 392.4 eV, respectively (Figure 4i),^[^ ^13a^ ^]^ respectively, indicating the formation of UO₂ on the surface of Cu_3_‐PA‐COF‐AA after the reaction.

The U(VI) wastewater generated from nuclear fuel production and nuclear power plant operations contains abundant F^−^ and various organics,^[^ ^37^ ^]^ which can compete with U(VI) to adsorb on the surface of photocatalysts. Therefore, their effect on the photocatalytic reduction of U(VI) in Cu_3_‐PA‐COF‐AA was explored. As shown in Figures S22 and S23 (Supporting Information), Cu_3_‐PA‐COF‐AA exhibits only a very slight decrease in the removal ratio of U(VI) even under a high F: U ratio of 16: 1 and in different organics such as tannic acid (TA), rhodamine B (RhB), bisphenol A (BPA) and methylene blue (MB). Additionally, considering the variation in U(VI) concentrations in groundwater across different regions, the effect of U(VI) concentration ranging from 1 to 300 ppm was investigated at a fixed sorbent concentration of 0.05 g L^−1^ (Figure S24, Supporting Information). The excellent photocatalytic activity was still retained under a wide initial U(VI) concentration. Furthermore, cation interference experiments demonstrate the negligible effect of various cations including Na^+^, K^+^, Ca^2+^, Zn^2+^, Sr^2+^, and Cu^2+^ (100 ppm) on photocatalytic activity of U(VI) reduction (Figure S25, Supporting Information).

### 2.4 Photocatalytic Mechanism Studies

**Figure 5:**
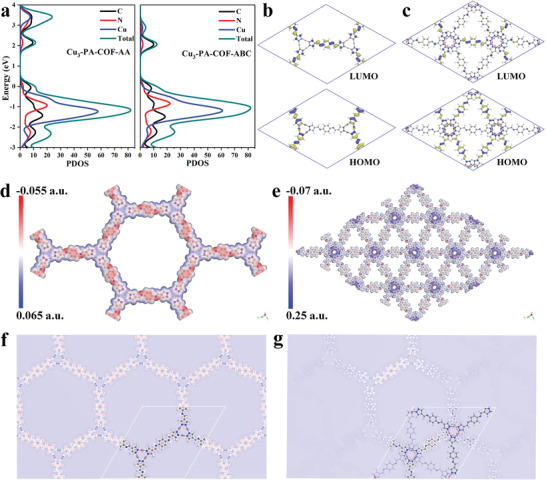
PDOS (a); HOMO and LUMO of Cu_3_‐PA‐COF‐AA (b) and Cu_3_‐PA‐COF‐ABC (c); ESP of Cu_3_‐PA‐COF‐AA (d) and Cu_3_‐PA‐COF‐ABC (e); Differential charge density diagram of Cu_3_‐PA‐COF‐AA (f) and Cu_3_‐PA‐COF‐ABC (g).

The energy band structures and partial density of states (PDOS) were obtained through theoretical calculation. As shown in Figure S26 (Supporting Information), in different k‐space regions, the valence band maximum (VBM) and the conduction band minimum (CBM) of Cu_3_‐PA‐COF‐AA and Cu_3_‐PA‐COF‐ABC both correspond to indirect bandgap semiconductors. The PDOS of the VBM is primarily contributed by the Cu and N orbitals (**Figure** **5**a), while the CBM has a greater contribution from C orbitals, indicating that the electron acceptor originates from PA and the electron donor from Cu_3_ units. This result is consistent with the molecular orbitals presented in Figure 5b,c, where the highest occupied molecular orbital (HOMO) is contributed by the Cu_3_ cluster and the lowest unoccupied molecular orbital (LUMO) by PA. Compared to Cu_3_‐PA‐COF‐ABC, Cu_3_‐PA‐COF‐AA exhibits more pronounced HOMO‐LUMO spatial separation, which promotes charge separation and decreases the recombination possibility.^[^ ^38^ ^]^ In addition, the electrostatic potentials (ESP) of two COFs indicate that the negative potential regions are primarily centered on the PA, while the positive potential regions are located on the Cu_3_ building block (Figure 5d,e). These results indicate electron transfer from Cu_3_ to PA upon excitation and the successful formation of an internal electric field between the two functional motifs in the COFs, consistent with the XPS results. Importantly, the ESP difference between Cu_3_ and PA in Cu_3_‐PA‐COF‐AA is more pronounced than that in Cu_3_‐PA‐COF‐ABC, indicating the more efficient charge transport in Cu_3_‐PA‐COF‐AA. Furthermore, as revealed by the PDOS, compared to VBM, CBM presents lower hybrid electron density, indicating the weak electrostatic attraction between the nucleus and electrons,^[^ ^39^ ^]^ which facilitates the electron transfer to the catalyst surface and hinders the charge recombination. Notably, the PDOS of the CBM in Cu_3_‐PA‐COF‐AA is significantly greater than in Cu_3_‐PA‐COF‐ABC, indicating that the electron acceptor in Cu_3_‐PA‐COF‐AA can accept more excited electrons,^[^ ^40^ ^]^ which enhances efficient charge transfer and the photoreduction of U(VI). In addition, as revealed by the interfacial charge density difference in Figure 5f,g, the electron accumulation is shown in the blue region for Cu_3_, and the electron depletion is presented in the red region for PA. Notably, the charge density difference between Cu_3_ and PA in Cu_3_‐PA‐COF‐AA is more pronounced than in Cu_3_‐PA‐COF‐ABC, which facilitates charge transport. These theoretical calculations further demonstrate the more effective transport and separation of photogenerated charge carriers in Cu_3_‐PA‐COF‐AA, which significantly enhances the photocatalytic reduction of U(VI) to U(IV).

## 3 Conclusion

In summary, we have successfully synthesized two trinuclear copper organic frameworks with distinct interlayer stacking modes (eclipsed AA stacking and staggered ABC stacking) using the same Cu_3_ and PA monomers. As expected, the two COFs exhibit distinctly different chemical and physical properties. Compared to Cu_3_‐PA‐COF‐ABC with staggered ABC stacking, Cu_3_‐PA‐COF‐AA with eclipsed AA stacking exhibits a broader visible‐light absorption range and more effective charge transfer and separation efficiency. Consequently, the photocatalytic activity of Cu_3_‐PA‐COF‐AA toward U(VI) reduction is significantly superior to that of Cu_3_‐PA‐COF‐ABC. A U(VI) removal ratio as high as 93.6% is achieved with Cu_3_‐PA‐COF‐AA without additional sacrificial agents in the air, ≈2.2 times higher than that achieved with Cu_3_‐PA‐COF‐ABC (42.0%). Furthermore, Cu_3_‐PA‐COF‐AA demonstrates excellent reusability, highlighting its significant potential for practical application in radioactive wastewater treatment. Our work demonstrates that modulating the interlayer stacking modes of 2D COFs can open new avenues for the rational design of highly effective photocatalysts for water pollution remediation, potentially stimulating further exploration of the relationship between the intrinsic structural properties of COFs and their photocatalytic performance.

## Conflict of Interest

The authors declare no conflict of interest.

## Supporting information



Supporting Information

## Data Availability

The data that support the findings of this study are available from the corresponding author upon reasonable request.
