# Supplementary material for: Precise Regulation of Interlayer Stacking Modes in Trinuclear Copper Organic Frameworks for Efficient Photocatalytic Reduction of Uranium(VI)
Source: Adv Sci (Weinh). 2024 Sep 27;11(43):2406530. doi: 10.1002/advs.202406530 (PMC11578321; doi:10.1002/advs.202406530)
Supplement: Supplementary file 1 — Supporting Information [file ADVS-11-2406530-s001.docx]

**Precise Regulation of Interlayer Stacking Modes in Trinuclear Copper Organic Frameworks for Efficient Photocatalytic Reduction of Uranium(VI)**

Zhi Gao^a^, Sijia Lv^a^, Yue Wang^a^, Zhenzhen Xu^a^*, Yingtong Zong^c^, Yuan Tao^d^, Yingji Zhao^e^*, Xingyu Liu^a^, Shuhui Yu^a^, Mingbiao Luo^a^, Nithima Khaorapapong^f^, Ruikang Zhang^b^*, Yusuke Yamauchi^e,g,h^

^a^ Jiangxi Province Key Laboratory of Functional Organic Polymers, East China University of Technology, Nanchang, Jiangxi 330013, China. E-mail: xu_zhenzhen@ecut.edu.cn

^b^ College of Chemistry and Materials Science, Hebei Normal University, Shijiazhuang, Hebei 050024, China. E-mail: zhangruikang@hebtu.edu.cn

^c^ College of Chemistry and Chemical Engineering, Gannan Normal University, Ganzhou, Jiangxi 341000, China.

^d^ MOE Key Laboratory of Bioinorganic and Synthetic Chemistry/KLGHEI of Environment and Energy Chemistry, School of Chemistry, Sun Yat-Sen University, Guangzhou, Guangdong 510275, China.

^e^ Department of Materials Process Engineering, Graduate School of Engineering, Nagoya University, Nagoya 464-8603, Japan. E-mail: yingji.zhao@akane.waseda.jp

^f^ Materials Chemistry Research Center, Department of Chemistry and Center of Excellence for Innovation in Chemistry, Faculty of Science, Khon Kaen University, Khon Kaen 40002, Thailand.

^g^ Department of Chemical and Biomolecular Engineering, Yonsei University, 50 Yonsei-ro, Seodaemun-gu, Seoul 03722, South Korea.

^h^ Australian Institute for Bioengineering and Nanotechnology (AIBN), The University of Queensland, Brisbane, QLD 4072, Australia.

**Experimental Section**

**Materials.** All reagents were used as received without further purification. 1H-pyrazole-4-carbaldehyde (1H-PyCA), Cu(NO_3_)_2_·3H_2_O, *p*-phenylenediamine (PA), acetic acid, and uranyl nitrate hexahydrate (UO_2_(NO_3_)_2_·6H_2_O) were purchased from the Shanghai Aladdin Biochemical Technology Co., LTD. Arsenazo III, N,N-dimethylformamide (DMF), ethanol, *p*-toluenesulfonic acid, tetrahydrofuran (THF), mesitylene, and 1,4-dioxane were obtained from Shanghai Macklin Biochemical Co., Ltd (China).

**Synthesis of Cu_3_:** Firstly, Cu(NO_3_)_2_·3H_2_O (0.83 mmol, 0.20 g) and 1H-PyCA (1.0 mmol, 0.096 g) were dissolved in the mixed solvents of DMF (6.7 mL), deionized water (5 mL) and ethanol (6.7 mL). Then, the homogeneous mixed solution was transferred into an autoclave (25 mL) with a Teflon liner, followed by heating at 100 ^o^C for 24 h. After the reaction, the light yellow single crystals obtained were immersed in deionized water for three days. In the meantime, the deionized water was exchanged 6 times per day. Finally, the crystals were washed quickly with acetone for three times, followed by drying at 120 ^o^C for 24 h under a vacuum, which were denoted as Cu_3_.

**Synthesis of Cu_3_-PA-COF-AA:** Firstly, *p*-phenylenediamine (PA, 0.2 mmol, 0.022 g) and *p*-toluenesulfonic acid (1.14 mmol, 0.22 g) were added into a mortar, followed by grinding for 5 min using a pestle. Then, Cu_3_ cluster (0.14 mmol，0.068 g) was put into the above solid mixture with constant grinding for another 10 min. Afterward, deionized water (60 μL) was added and ground again for 5 min. After standing for 1 h at room temperature, the mixture was transferred to a crucible placed in an oven, followed by heating at 170 ^o^C for 15 min. In order to remove the unreacted *p*-toluenesulfonic acid and PA in the as-obtained sample, it was immersed in deionized water for three days, during which the deionized water was exchanged 6 times per day by centrifugation. Finally, the solvent exchange with acetone (6 times per day for 3 days) was performed and then dried under vacuum at 80 ^o^C for 12 h, which is denoted as Cu_3_-PA-COF-AA.

**Synthesis of Cu_3_-PA-COF-ABC:** PA (0.1 mmol, 0.011 g) and Cu_3_ cluster (0.07 mmol, 0.034 g) were mixed in a cylindrical glass tube (10 cm of length, ф_in_ = 2.2 cm, ф_out_ = 2.6 cm) and then mesitylene (2 mL), 1,4-dioxane (2 mL) and 6 M aqueous acetic acid (0.4 mL) were added. Afterward, the mixture was sonicated to get a homogeneous dispersion and then flash frozen at 77 K in a liquid nitrogen bath and degassed with three freeze-pump-thaw cycles. Upon warming to room temperature, the mixture was heated at 120 °C for 72 h. The as-prepared crystalline powder was washed with DMF to remove the unreacted Cu_3_ and PA. Then, the sample was transferred to a Soxhlet extractor and washed with THF for 24 hours. Finally, the product was dried under a vacuum at 120 ^o^C for 12 h.

**Photocatalytic reduction of U(VI):** Firstly, a particular amount of UO_2_(NO_3_)_2_·6H_2_O was dissolved in deionized water to prepare the U(VI) solutions. Then, the photocatalytic activity toward U(VI) reduction was performed in a photoreactor (200 mL) without any sacrificial agents under visible light irradiation in the air atmosphere using a 300 W xenon lamp (PerfectLight, PLS-SXE300D) with a 420 nm cut-off filter. Briefly, the sample (5 mg) and the U(VI) solution (100 mL, 20 mg/L) were added into a glass reaction vessel. After a reaction of a certain time, aliquots of the dispersion were collected and filtered through a 0.45 μm membrane filter. In order to test the reusability, the photocatalyst was collected after the photocatalytic experiment and dried sufficiently, which was then subjected to a solution of 0.1 M HCl (20 mL) for 3 h. The dilute hydrochloric acid solution was refreshed every hour. After that, the deionized water was used to wash the photocatalyst for the next cycle. The concentration of U(VI) in the filtrates was determined by UV-vis spectrophotometry using the Arsenazo III method at a wavelength of 652 nm. In addition, the U(VI) adsorption tests were conducted in similar procedures except that the Xenon lamp was not used for illumination.

The removal ratio, removal capacity (*q*_t_, mg g^-1^), and photoreaction rate constant (*k*_t_) were calculated using the following equation.

Removal ratio = (*C*_0_ - *C*_t_)/*C*_0_ × 100%

*q*_t_ = (*C*_0_ - *C*_t_) × V/m

*k*_t_ = ln (*C*_0_/*C*_t_)

Where *C*_t_ is the time concentrations of U(VI) (mg L^-1^), *C*_0_ is the initial concentrations of U(VI) (mg L^-1^), V is the volume of the solution (L), t is the reaction time (min), m is the COF mass (g).

**Characterization:** Powder X-ray diffraction (PXRD) data was collected on a Rigaku SmartLab SE X-ray diffractometer. Solid-state ^13^C CP/MAS NMR measurements were taken on a Bruker AVANCE III 400 WB spectrometer. Brunauer-Emmett-Teller (BET) method was utilized to calculate the specific surface areas and pore size on a Micromeritics ASAP2020 analyzer at 77 K. The pore size distribution curves were obtained by non-local density functional theory (NLDFT). Fourier transform infrared spectra (FT-IR) were recorded on a Bruker VERTEX70 spectrometer. Ultraviolet-visible diffuse reflectance spectra (UV-vis DRS) were recorded from 200-800 nm on a Shimadzu corporation U-2700 spectrophotometer. Electron paramagnetic resonance (EPR) spectra were recorded on a Bruker EPR A300 spectrometer. Photoelectrochemical experiments were performed on a CHI660E workstation. X-ray photoelectron spectroscopy (XPS) measurements were carried out on a Theta probe (Thermo Fisher) with Al Kα X-rays. Scanning electron microscopy (SEM) images were obtained on the Oxford X-max microscope. High-resolution transmission electron microscopy (HRTEM) was carried out on a Tecnai F20 at 200 KV. Steady-state photoluminescence (PL) and fluorescence decay spectra were measured on FLS1000-Edinburgh Instruments. The thermogravimetric analysis (TGA) measurements were performed from 20 to 800 °C on a TA Q500 thermogravimeter.

**Computational details:** Structural modeling and Pawley refinement were carried out in the Materials Studio 2022 software package for crystal determination from PXRD patterns. The theoretical models were then optimized by the Forcite module. Pawley refinements of the PXRD patterns were performed in the Reflex module from 2° to 40°, which are agreeable with the simulated patterns of AA and ABC stacking models. Density functional theory (DFT) calculations were carried out using the CASTEP and DMol3 modules in the Materials Studio 2022 software package. The structural modeling was optimized at the method of Perdew-Burke-Ernzerhof (PBE) function under the generalized gradient approximation (GGA) functional by the CASTEP module. Ultrasoft pseudopotentials from the Pseudo-dojo project were used and the energy cutoff was 400 eV. A 1×1×3 grid was performed for Brillouin zone sampling. Then, the band structure, density of state, and electron density difference were also calculated at the same level by the CASTEP module, and the front molecular orbital and electrostatic potential were calculated at the method of Perdew-Burke-Ernzerhof (PBE) function under the generalized gradient approximation functional with DND basis set by DMol3 module.


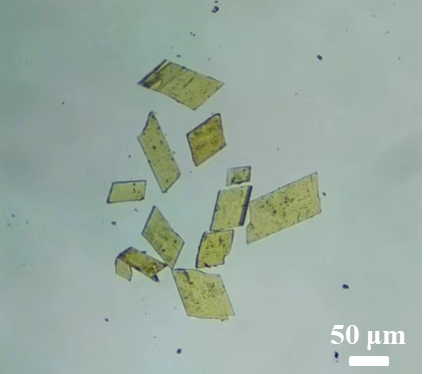


**Figure S1.** Optical microscope image of Cu_3_ cluster.





**Figure S2.** PXRD pattern of Cu_3_ cluster.





**Figure S3.** FT-IR spectra of 1H-PyCA and Cu_3_.





**Figure S4.** ^13^C CP/MAS NMR spectrum of Cu_3_-PA-COF-ABC.





**Figure S5.** XPS survey spectra of Cu_3_, Cu_3_-PA-COF-AA, and Cu_3_-PA-COF-ABC.





**Figure S6.** XPS spectra of C 1s.





**Figure S7.** XPS spectra of O 1s.


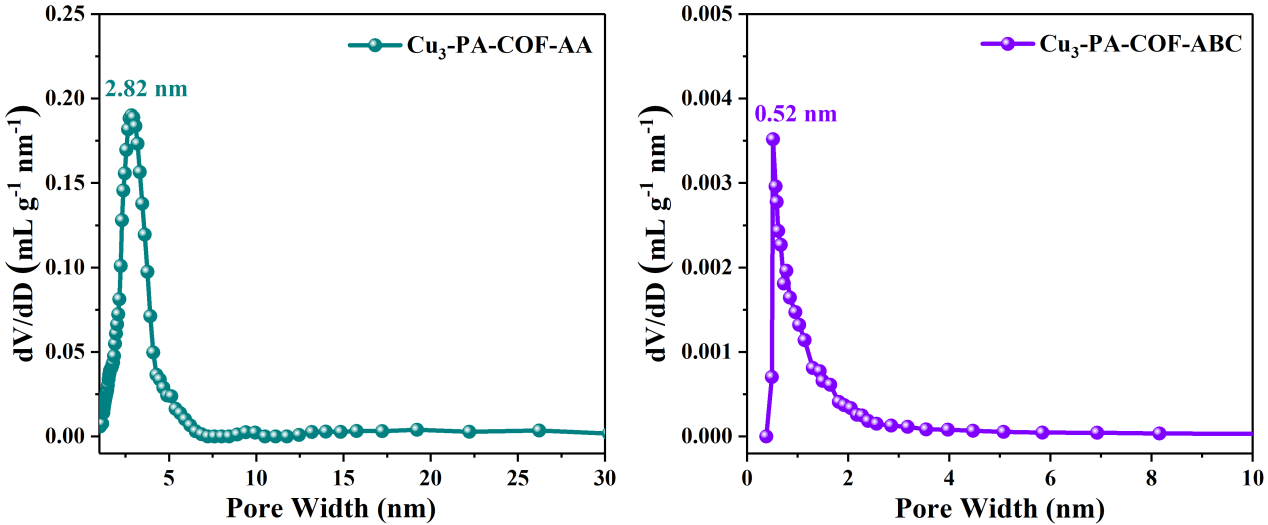


**Figure S8.** The pore size distribution of Cu_3_-PA-COF-AA**.**





**Figure S9.** TGA curves of Cu_3_-PA-COF-AA and Cu_3_-PA-COF-ABC**.**

**
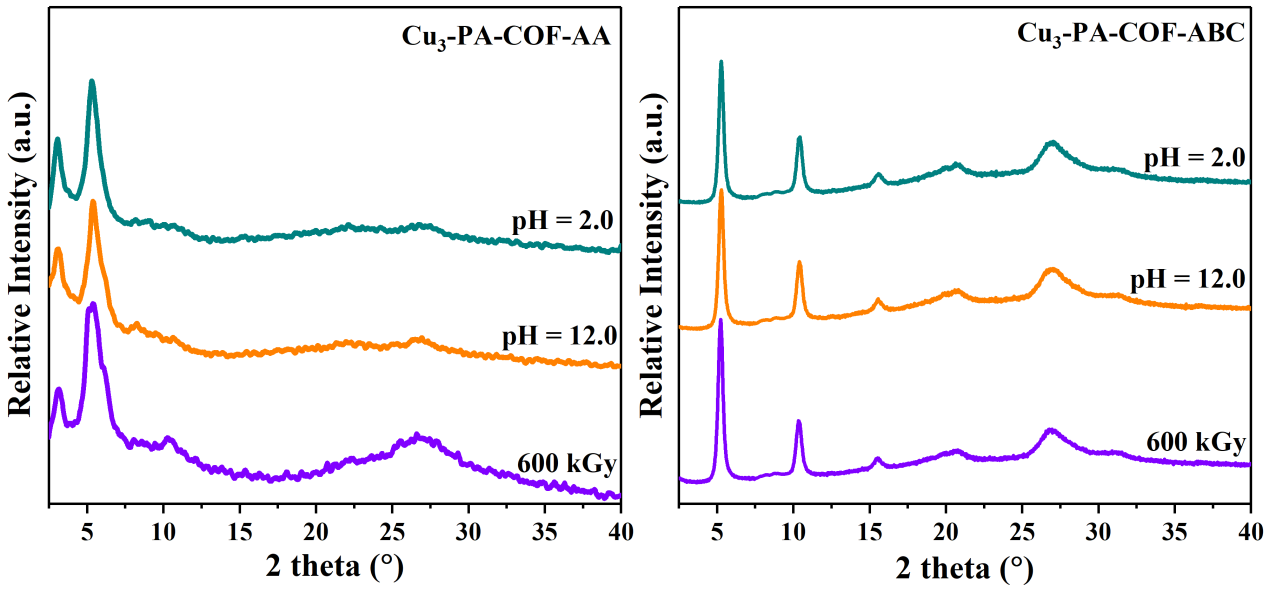
**

**Figure S10.** PXRD patterns of Cu_3_-PA-COF-AA and Cu_3_-PA-COF-ABC immersed in aqueous acid (pH = 2.0) and alkaline (pH = 12.0) solutions for 24 h and exposed in *β*-irradiation (600 kGy).


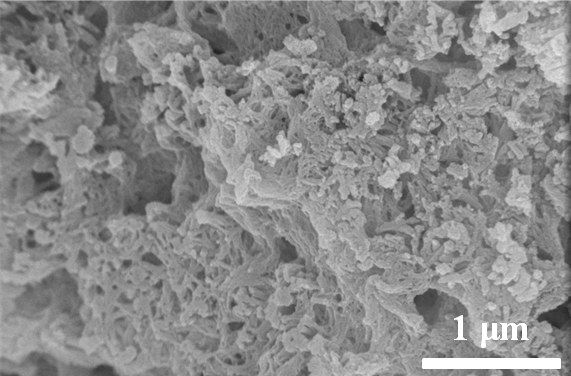


**Figure S11.** SEM image of Cu_3_-PA-COF-ABC**.**


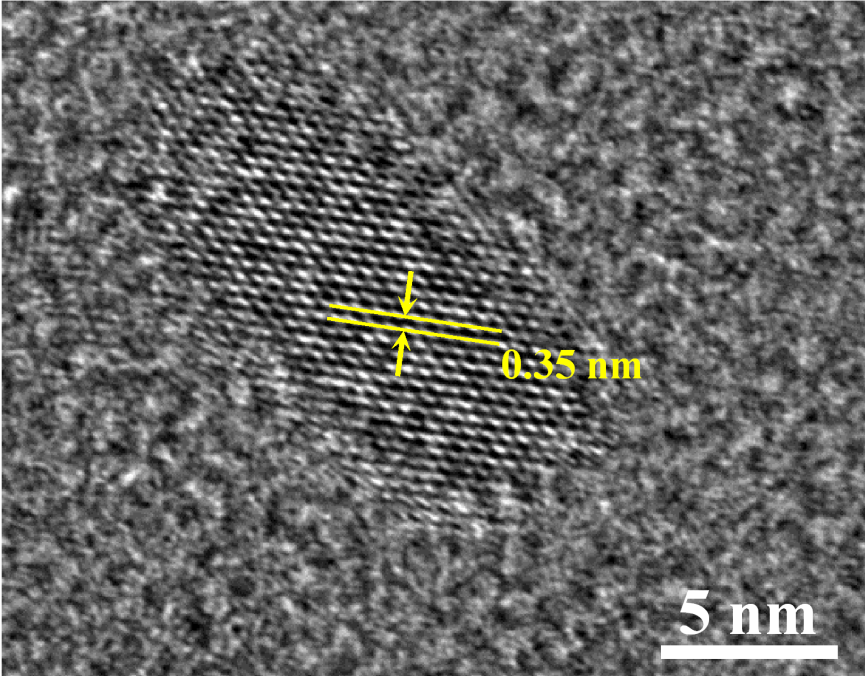


**Figure S12.** HRTEM image of Cu_3_-PA-COF-AA**.**


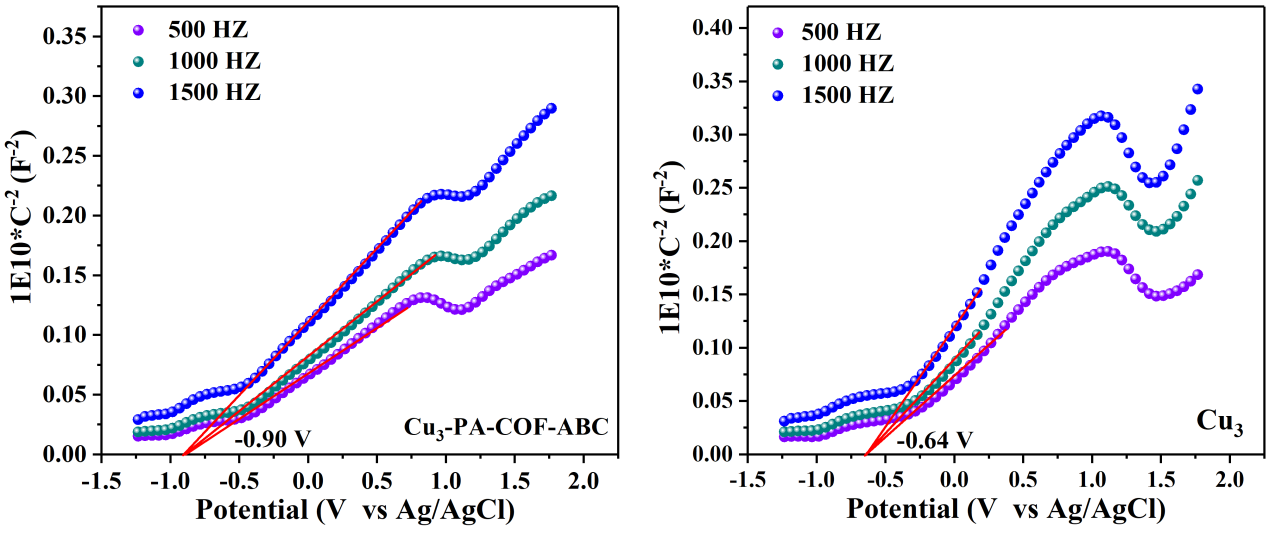


**Figure S13.** Mott-Schottky plots.





**Figure S14.** CV curve of Cu_3_-PA-COF-AA.

**

**

**Figure S15.** U(VI) removal capacity with the initial concentration of ~20 ppm.

**

**

**Figure S16.** Reaction kinetics plots of U(VI) photoreduction.





**Figure S17.** Removal ratio of Cu_3_-PA-COF-AA under N_2_ atmosphere.





**Figure S18.** Removal ratio of Cu_3_-PA-COF-AA versus different scavengers.





**Figure S19.** XPS spectra of O 1s in Cu_3_-PA-COF-AA before and after five cycles.





**Figure S20.** XPS spectra of C 1s in Cu_3_-PA-COF-AA before and after five cycles.





**Figure S21.** XPS survey spectra of Cu_3_-PA-COF-AA before and after five cycles.





**Figure S22.** Removal ratio of Cu_3_-PA-COF-AA in the U(VI) solution with different F^-^ concentrations.





**Figure S23.** Removal ratio of Cu_3_-PA-COF-AA in the U(VI) solution with different organics.





**Figure S24.** Removal ratio of Cu_3_-PA-COF-AA versus initial U(VI) concentration.





**Figure S25.** Removal ratio of Cu_3_-PA-COF-AA versus various cations.


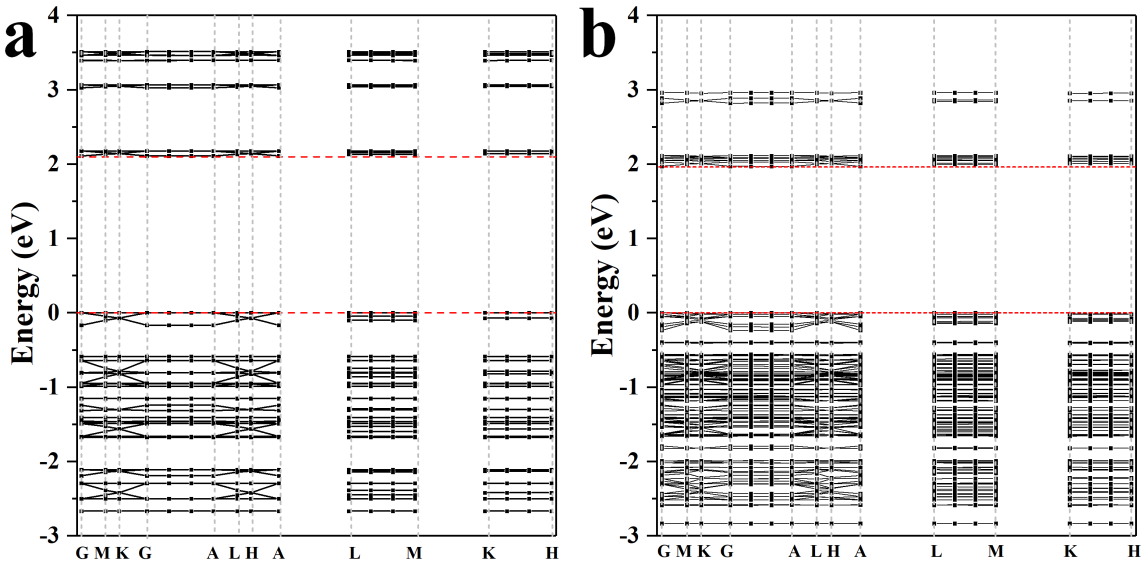


**Figure S26.** Calculated band structures of Cu_3_-PA-COF-AA (a) and Cu_3_-PA-COF-ABC (b).

**Table S1.** Comparison of the structural properties between Cu_3_-PA-COF-AA/Cu_3_-PA-COF-ABC and FDM-71^[1]^/FDM-71-ABC^[2]^.

| **Structural properties** | **Sample** | | | |
| --- | --- | --- | --- | --- |
|  | Cu_3_-PA-COF-AA | FDM-71^[1]^ | Cu_3_-PA-COF-ABC | FDM-71-ABC^[2]^ |
| Light absorption ability | Strong visible-light absorption | Strong visible-light absorption | Strong visible-light absorption | Strong visible-light absorption |
| Thermal stability | 300 ^o^C | 270 ^o^C | 280 ^o^C | -- |
| Optimal band gap | 2.20 eV | 2.09 eV | 2.45 eV | 2.71 eV |
| Conduction band | -0.66 V vs. NHE | -- | -0.70 V vs. NHE | -0.76 V vs. NHE |
| BET surface area | 496.6 m^2^ g^-1^ | 1135 m^2^ g^-1^ | 24.56 m^2^ g^-1^ | -- |
| Pore size | 2.82 nm | 2.0-3.5 nm | -- | -- |

**Table S2.** The different catalysts for U(VI) photoreduction in recent literature.

| Photocatalysts | U(VI)  concentration | Solid-to-liquid ratio (g/L) | Electron  sacrifice | Time  (min) | Removal rate (%) | Ref. |
| --- | --- | --- | --- | --- | --- | --- |
| Cu_3_-PA-COF-AA | 20 ppm | 0.05 | none | 390 | 93.6 | This work |
| SnS_2_COF | 550 ppm | 0.5 | none | 120 | 98.5 | [3] |
| PTrSO-2 | 50 ppm | 0.5 | methanol | 120 | 99.5 | [4] |
| PMo_12_/UiO-66 | 50 ppm | 0.2 | methanol | 300 | 98.92 | [5] |
| TpTt | 30 ppm | 0.229 | methanol | 480 | 55 | [6] |
| BiOBr@TpPa-1 | 30 ppm | 0.333 | methanol | 540 | 91 | [7] |
| TT-Por COF-Ni | 200 ppm | 1 | none | — | 83.0 | [8] |
| TpPa-COOH | 100 ppm | 0.167 | methanol | 420 | 77.29 | [9] |
| pTTT-Ben | 50 ppm | 1 | ascorbic acid | 240 | 78 | [10] |
| TiOS/NHCS | 10 ppm | 0.4 | ethanol | 20 | 90 | [11] |
| BCN-80 | 1 mM | 5 | isopropanol | 90 | 97.4 | [12] |
| DQTP | 30 ppm | 0.167 | methanol | 300 | 64 | [13] |

**References**

[1] X. Li, J. Wang, F. Xue, Y. Wu, H. Xu, T. Yi, Q. Li, *Angew. Chem. Int. Ed.* **2021**, *60*, 2534.

[2] J. Zhou, J. Li, L. Kan, L. Zhang, Q. Huang, Y. Yan, Y. Chen, J. Liu, S.-L. Li, Y.-Q. Lan, *Nat. Commun.* **2022**, *13*, 4681.

[3] X. Liu, R.-X. Bi, C.-R. Zhang, Q.-X. Luo, R.-P. Liang, J.-D. Qiu, *Chem. Eng. J.*, **2023**, *460*, 141756.

[4] F. Yu, Z. Zhu, S. Wang, J. Wang, Z. Xu, F. Song, Z. Dong, Z. Zhang, *Appl. Catal. B: Environ.*, **2022**, *301*, 120819.

[5] Z. Zhang, Z. Li, Z. Dong, F. Yu, Y. Wang, Y. Wang, X. Cao, Y. Liu, Y. Liu, *Chinese Chem. Lett.*, **2022**, *33*, 3577-3580.

[6] X. Zhong, Q. Ling, Z. Ren, B. Hu, *Appl. Catal. B: Environ.*, **2023**, *326*, 122398.

[7] X. Zhong, Y. Liu, S. Wang, Y. Zhu, B. Hu, *Sep. Purif. Technol.*, **2021**, *279*, 119627.

[8] L. Chen, J. Hang, B. Chen, J. Kang, Z. Yan, Z. Wang, Y. Zhang, S. Chen, Y. Wang, Y. Jin, C. Xia, *Chem. Eng. J.*, **2023**, *454*, 140378.

[9] X. Zhong, Q. Ling, P. Kuang, B. Hu, *Chem. Eng. J.*, **2023**, *467*, 143415.

[10] B. Chen, G. Zhang, L. Chen, J. Kang, Y. Wan, S. Chen, Y. Jin, H. Yan, C. Xia, *J. Hazard. Mater.*, **2022**, *426*, 127851.

[11] H. Wan, Y. Li, M. Wang, Q. Zhao, Y. Fu, Y. Chen, P. He, L. Wu, Q. Meng, T. Ma, J. Yang, T. Duan, *Chem. Eng. J.*, **2022**, *430*, 133139.

[12] Y. Wang, G. Chen, H. Weng, L. Wang, J. Chen, S. Cheng, P. Zhang, M. Wang, X. Ge, H. Chen, W. Huang, M. Lin, *Chem. Eng. J.*, **2021**, *410*, 128280.

[13] Q. Ling, P. Kuang, X. Zhong, B. Hu, *Appl. Surf. Sci.*, **2023**, *639*, 158220.
